# Establishment of Inducible Wild Type and Mutant Myocilin-GFP-Expressing RGC5 Cell Lines

**DOI:** 10.1371/journal.pone.0047307

**Published:** 2012-10-17

**Authors:** Hongyu Ying, Xiang Shen, Beatrice Y. J. T. Yue

**Affiliations:** Department of Ophthalmology and Visual Sciences, University of Illinois at Chicago, College of Medicine, Chicago, Illinois, United States of America; Wayne State University, United States of America

## Abstract

**Background:**

Myocilin is a gene linked directly to juvenile- and adult-onset open angle glaucoma. Mutations including Gln368stop (Q368X) and Pro370Leu (P370L) have been identified in patients. The exact role of myocilin and its functional association with glaucoma are still unclear. In the present study, we established tetracycline-inducible (Tet-on) wild type and mutant myocilin-green fluorescence protein (GFP) expressing RGC5 stable cell lines and studied the changes in cell migration and barrier function upon induction.

**Methodology/Principal Findings:**

After several rounds of selection, clones that displayed low, moderate, or high expression of wild type, Q368X or P370L myocilin-GFP upon doxycycline (Dox) induction were obtained. The levels of wild type and mutant myocilin-GFP in various clones were confirmed by Western blotting. Compared to non-induced controls, the cell migration was retarded, the actin stress fibers were fewer and shorter, and the trypsinization time needed for cells to round up was reduced when wild type or mutant myocilin was expressed. The barrier function was in addition aberrant following induced expression of wild type, Q368X or P370L myocilin. Immunoblotting further showed that tight junction protein occludin was downregulated in induced cells.

**Conclusions/Significance:**

Tet-on inducible, stable RGC5 cell lines were established. These cell lines, expressing wild type or mutant (Q368X or P370L) myocilin-GFP upon Dox induction, are valuable in facilitating studies such as proteomics, as well as functional and pathogenesis investigations of disease-associated myocilin mutants. The barrier function was found impaired and the migration of cells was hindered with induced expression of wild type and mutant myocilin in RGC5 cell lines. The reduction in barrier function might be related to the declined level of occludin. The retarded cell migration was consistent with demonstrated myocilin phenotypes including the loss of actin stress fibers, lowered RhoA activities and compromised cell-matrix adhesiveness.

## Introduction

Glaucoma is a major blinding disease characterized by progressive loss of retinal ganglion cells (RGCs) and their axons, as well as cupping of the optic nerve head. The most common form of this disease, primary open angle glaucoma (POAG), is highly heterogeneous, caused by several susceptibility genes [1] and perhaps also environmental factors [2]. To date, candidate genes including myocilin as GLC1A [3], [4] and optineurin as GLC1E [5], [6] have been identified.

Myocilin, the first candidate gene linked to juvenile- and adult-onset POAG, was originally cloned from cultured human trabecular meshwork (TM) cells after prolonged treatment of dexamethasone [7], [8]. The human myocilin gene encodes an acidic glycoprotein of 504 amino acids (aa). Sequence analysis has revealed an amino (N)-terminal coiled coil domain (also known as nonmuscle myosin-like domain) containing therein a leucine zipper motif [9], a signal sequence that targets myocilin for secretion [10], a central linker region, and a carboxyl (C)-terminal olfactomedin-like domain. Mutations of myocilin were found in 2–4% of POAG patients. More than 70 mutations in myocilin have been reported [2], [11]. The disease-causing ones among them are located predominantly in the olfactomedin-like domain [12]. Gln368Stop (Q368X) is the most common myocilin mutation reported in POAG patients (with occurrence of about 1.6%) [12]. With nonsense mutation at aa residue 368, it generates a truncated protein of 367 aa length. Pro370Leu (P370L), a missense mutation, is responsible for one of the most severe glaucoma phenotypes [13], [14], [15]. http://www.sciencedirect.com/science/article/pii/S0002944010603501 - ref_bib13

Myocilin protein is detected in eye tissues including the TM, the Schlemm's canal, the sclera, the ciliary body, the retina and the optic nerve head [16], [17]. It interacts with itself and a number of other proteins, mainly through the leucine zipper motif and the coiled coil region in the myosin-like domain [18], [19], [20]. The wild type myocilin is a secreted protein [7], [21], [22]. Mutants with mutations in the olfactomedin-like domain, however, are not secreted. They are retained in the cells, aggregating to cause endoplasmic reticulum stress and unfold protein response [23], [24], [25].

To facilitate studies of myocilin and its mutants, we established tetracycline-inducible (Tet-on) RGC5 stable cell lines that would express, upon induction, green fluorescence protein (GFP)-tagged wild type and mutant (Q368X and P370L) myocilin. These cell models allowed studies that require confluent myocilin-expressing cell cultures such as migration and barrier functions. Our results disclosed that when the expression of wild type or mutant myocilin was induced, the actin stress fibers were lost, RhoA activity was reduced and cell migration was blocked. In addition, the trypsinization sensitivity was heightened and the barrier function was impaired. The expression level of tight junction protein occludin was also lowered which may contribute to the reduced barrier function.

## Results

### Establishment of tetracycline inducible (Tet-on) wild type and mutant myocilin-GFP RGC5 stable cell lines

**Figure 1:**
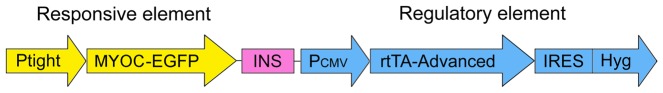
Single plasmid construct with two expression cassettes. The two cassettes (responsive element, in yellow, and regulatory element, in blue) are separated by 5′-HS4 chicken β-globin insulator (INS, in pink).

The inducible Tet-on wild type myocilin-GFP (myocilin_WT_-GFP or MYOC_WT_-GFP) expressing RGC5 stable cell lines were established using a single plasmid vector pTRE-MYOC-EGFP-INS-rtTA-IRES-hyg-pcDNA3.1z, which contains both tetracycline regulatory and responsive components based on Clontech's Tet-on advance system (Fig. 1). When transfected into RGC5 cells, myocilin_WT_-GFP was expressed following doxycycline (Dox) induction.

**Figure 2:**
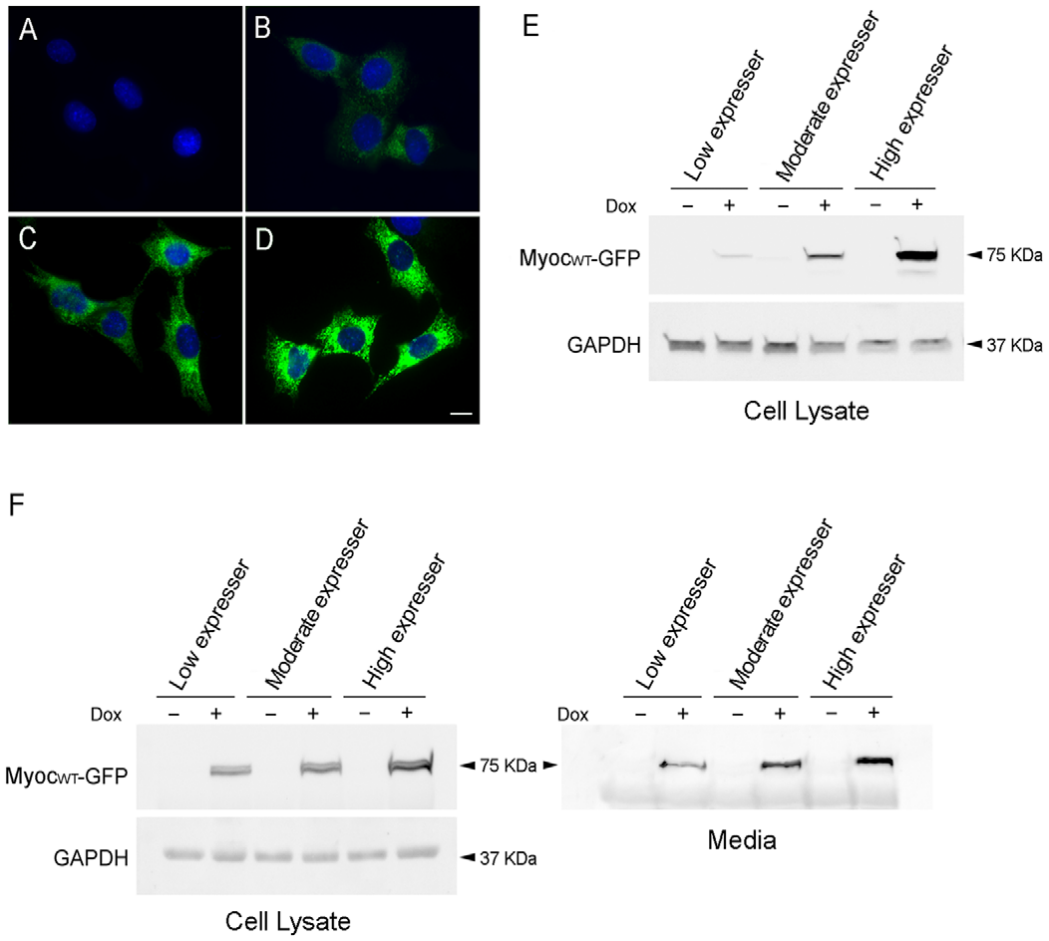
Tet-on inducible RGC5 cell lines that express wild type myocilin-GFP upon Dox induction. Clones with different expression levels of myocilin-GFP (MYOC_WT_-GFP) fusion protein are presented. Without Dox induction, the green fluorescence in the cells, indicative of MYOC_WT_-GFP expression, was minimal at a background level (**A**). After Dox treatment, low (**B**), moderate (**C**) and high (**D**) levels of MYOC_WT_-GFP were seen in, respectively, low, moderate, and high expresser clones. MYOC_WT_-GFP had a more spread out, cytoplasmic distribution pattern similar to that of the endogenous myocilin in the low expresser. Accumulation of MYOC_WT_-GFP was seen in high expresser clones. Scale bar, 10 µm. **E.** Western blot analyses of cell lysates using polyclonal anti-GFP and anti-GAPDH antibodies. **F**. Western blotting of cell lysates (left panel) and media samples (right panel) using monoclonal anti-myocilin antibody. Results in **E** and **F** confirmed that the level of MYOC_WT_-GFP relative to that of GAPDH in total cell lysates was low, moderate, and high from the various expresser clones. MYOC_WT_-GFP fusion protein was also detected in the culture media (**F**, right panel) with anti-myocilin antibody. −, Non-induced control; +, Induced cells.

After several rounds of selection, clones with different expression levels of wild type myocilin-GFP were obtained. When non-induced with no Dox present, only background fluorescence was detected in the cells (Fig. 2A). After Dox induction, the low expresser exhibited faint (Fig. 2B), while the moderate (Fig. 2C) and the high (Fig. 2D) expressers showed stronger green fluorescence in the cytoplasm. Microscopic examination indicated that myocilin_WT_-GFP in the low expresser had a more spread out, cytoplasmic distribution pattern similar to that of the endogenous myocilin. Cytoplasmic granules or aggregates, in varying extents, were observed in the moderate and high expressers. Western blotting using both anti-GFP (Fig. 2E) and anti-myocilin (Fig. 2F) antibodies confirmed that the induced level of myocilin_WT_-GFP was low in the low expresser but moderate or high in the others. As a secreted protein [7], [10], [21], [22], myocilin_WT_-GFP was detected in medium samples from induced cultures (Fig. 2F) as anticipated.

**Figure 3:**
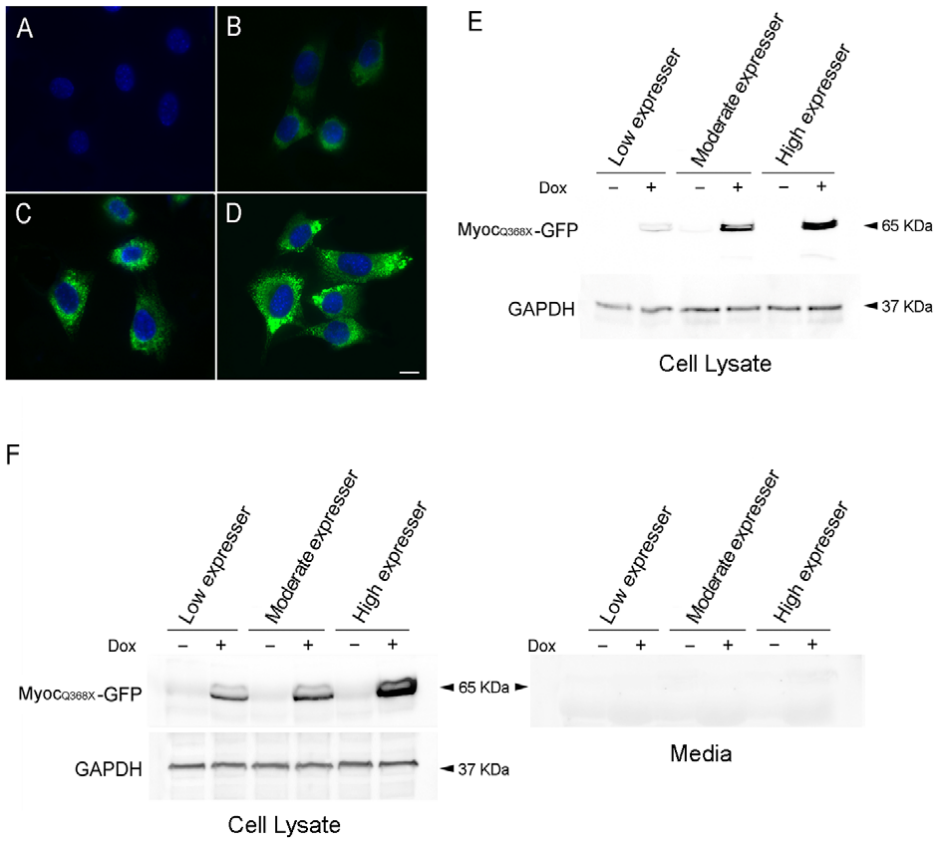
Tet-on inducible RGC5 cell lines that express mutant Q368X myocilin-GFP upon Dox induction. Clones with different expression levels of Q368X myocilin-GFP (MYOC_Q368X_-GFP) fusion protein are presented. Without Dox induction, the green fluorescence in the cells was minimal (**A**). After Dox treatment, low (**B**), moderate (**C**) and high (**D**) levels of MYOC_Q368X_-GFP were seen in respectively, low, moderate, and high expresser clones. Cytoplasmic aggregates were visible in moderate and high expressers. Scale bar, 10 µm. **E.** Western blot analyses using anti-GFP and anti-GAPDH polyclonal antibodies. **F**. Western blot analyses of cell lysate (left panel) and medium (right panel) samples using anti-myocilin monoclonal antibody. The blots confirmed that the level of MYOC_Q368X_-GFP relative to that of GAPDH in total cell lysates was low, moderate, and high from the various expresser clones. No MYOC_Q368X_-GFP protein band was observed in medium samples. −, Non-induced control; +, Induced cells.

**Figure 4:**
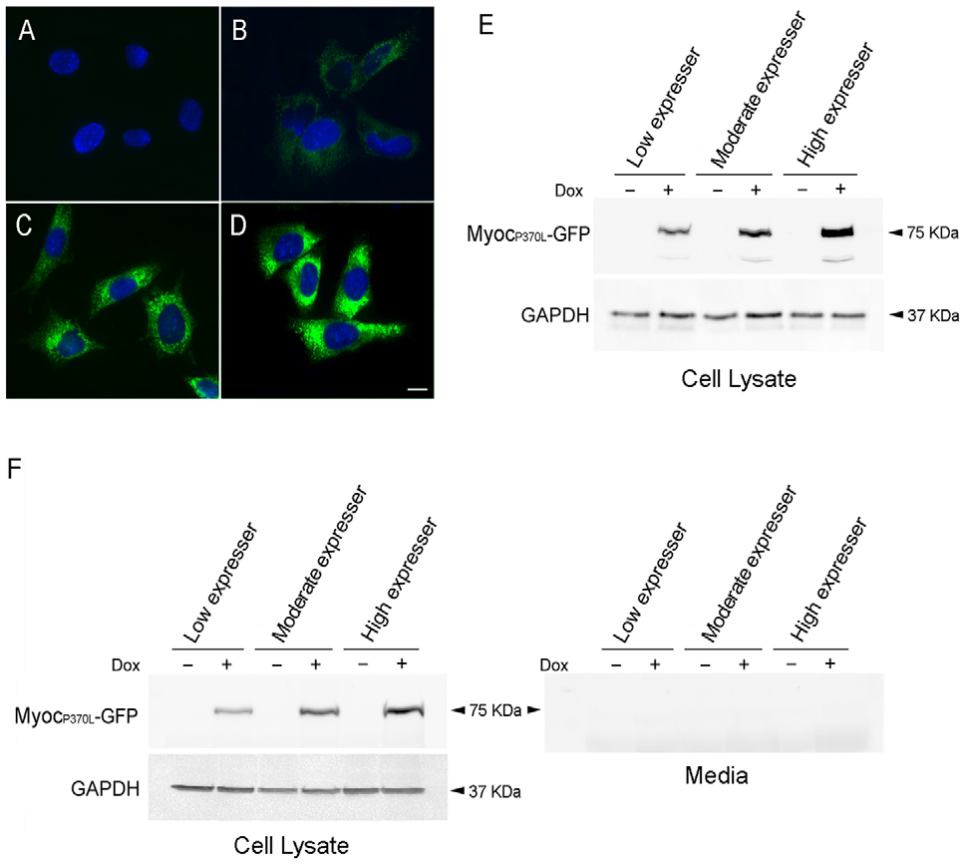
Tet-on inducible RGC5 cell lines express mutant P370L myocilin-GFP upon Dox induction. Clones with different expression levels of P370L myocilin-GFP (MYOC_P370L_-GFP) fusion protein are presented. Without Dox induction, the green fluorescence in the cells was minimal (**A**). After Dox treatment, low (**B**), moderate (**C**) and high (**D**) levels of MYOC_P370L_-GFP were seen in, respectively, low, moderate, and high expresser clones. Cytoplasmic aggregates were visible in both moderate and high expressers. Scale bar, 10 µm. **E.** Western blot analyses of cell lysates using anti-GFP and anti-GAPDH polyclonal antibodies. **F**. Western blot analyses of lysate (left panel) and media samples (right panel) using anti-myocilin monoclonal antibody. The blots confirmed that the level of MYOC_P370L_-GFP relative to that of GAPDH in total cell lysates was low, moderate, and high from the various expresser clones. No MYOC_P370L_-GFP protein band was seen in medium samples. −, Non-induced control; +, Induced cells.

Inducible cell lines that expressed two mutant myocilin (myocilin_Q368X_ or myocilin_P370L_)-GFP were additionally prepared using a similar strategy. Clones with different expression levels (low, moderate, and high) were also obtained and banked in liquid nitrogen. Myocilin_Q368X_-GFP in the low expressers displayed a diffuse cytoplasmic distribution pattern (Fig. 3B). In the moderate and high expressers, aggregates in the cytosol of the cells were evident (Fig. 3C, D). Likewise, cytoplasmic aggregation was observed in moderate and high expressers of myocilin_P370L_-GFP (Figs. 4B–D). Again, in non-induced clones, the fluorescence signal was minimal (Figs. 3A and 4A). The fusion protein expression level in various clones was verified by Western blotting probed with both anti-GFP (Figs. 3E and 4E) and anti-myocilin (Figs. 3F and 4F) antibodies. No mutant fusion protein was detected in the media collected from the various cultures, consistent with the notion that Q368X and P370L mutants were secretion incompetent [23], [24].

### Inhibition of cell migration when wild type and mutant myocilin-GFP was expressed

**Figure 5:**
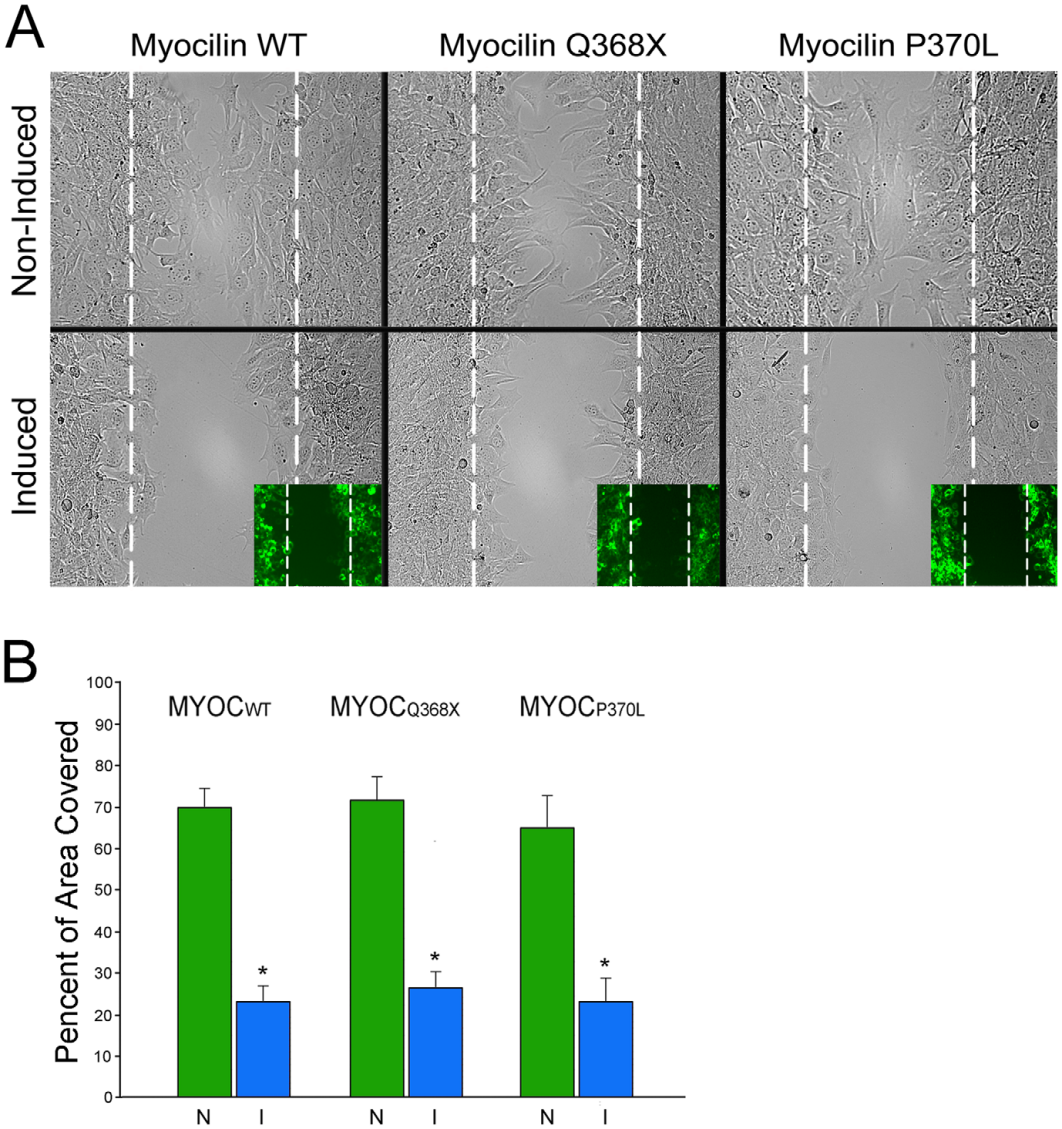
**A.** *In vitro* scratch assays. Cell migration was inhibited when moderate expressers were induced to express myocilin wild type (WT), Q368X, or P370L-GFP (lower panel) compared with non-induced controls (top panel). Inset shows the same view under a fluorescence microscope. The induced cells are in green. Scale bar, 50 µm. **B.** Bar graph to show the percent area (mean ± SD) covered by myocilin (MYOC)_WT_-, MYOC_Q368X_-, or MYOC_P370L_-GFP-expressing cells that were migrated into the scratched area. N, Non-induced control; I, Induced cells. *, P<0.0001 compared to non-induced control.

Cell migration was examined using an *in vitro* scratch assay. RGC5 cells were serum starved, treated with 5 µg/ml of mitomycin C for 2 h [26] and subjected to scratch assay in the presence of mitomycin C to block cell proliferation. It was found that compared to non-induced controls (Fig. 5A, upper panel), moderately induced expression of myocilin_WT_-, myocilin_Q368X_ -, or myocilin_P370L_-GFP caused a marked decrease in the ability of RGC5 cells to migrate (Fig. 5A, lower panel). At the 11 h post scratch time point, the percent of scratched area covered by migratory cells was reduced by 2–3 fold in the induced cultures (Fig. 5B, values for the wild type myocilin, non-induced [N]: 70.1±6.2%, induced [I]: 23.0±4.0%; for Q368X myocilin, N: 72.3±7.7%, I: 27.1±7.6%; for P370L myocilin, N: 64.6±8.3%, I: 25.3±7.5%; n = 8). The differences between non-induced and induced samples were statistically significant (P<0.0001).

### Loss of actin stress fibers, increased trypsin sensitivity, and lowered RhoA activities

**Figure 6:**
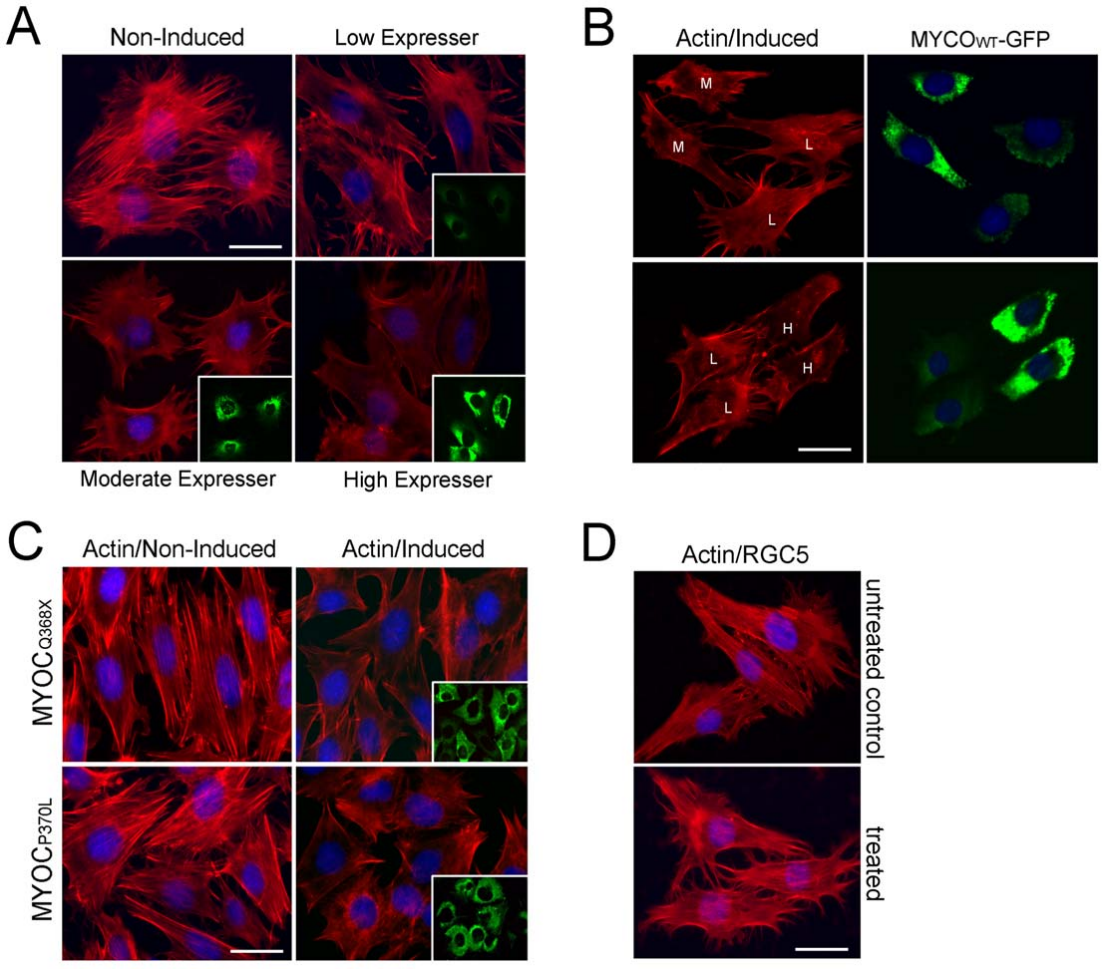
Actin staining in inducible RGC5 cells. **A**. Non-induced control cells, as well as low, moderate and high expressers of MYOC_WT_-GFP were stained with rhodamine-phalloidin for actin stress fibers. Compared to non-induced cells, the actin staining (red) was much reduced in Dox-induced ones. Insets show the same view of induced cells under FITC filter (green for myocilin-GFP expression). Scale bar, 10 µm. The loss of actin stress fibers, most dramatically observed in high expresser, was dose-dependent. **B**. Actin staining (red, left column) in mix cultures of low (L) and moderate (M) (top panel) or low (L) and high (H) (bottom panel) expressers was performed. The right column shows the same view with FITC filter to depict the green fluorescent transgene level. The stronger the green fluorescence, the more dramatic was the loss of actin fibers. **C**. Compared to non-induced controls (left column), Q368X- and P370L-GFP expression in induced cells (moderate expressers, right column) also caused a loss of actin stress fibers (red). Insets show the same view of induced cells under FITC filter. **D**. Robust actin stress fibers (red) were observed in parental RGC5 cells without (untreated control, top panel) or with Dox (1 µg/ml, bottom panel) treatment. Scale bar, 20 µm.

By phalloidin staining, a loss of actin stress fibers was observed when RGC5 cells were induced to express wild type myocilin at low, moderate or high levels (Fig. 6A). This result was in agreement with our previous study, in which myocilin transfection was shown to result in reduction of actin stress fibers and focal adhesions in human TM cells [27], [28]. The loss of actin stress fibers was dose-dependent (Fig. 6A). When low expresser was mix-cultured with moderate or high expresser and induced together, the actin loss was more dramatically observed in cells that displayed stronger green fluorescence with higher levels of myocilin-GFP expression (Fig. 6B). A loss of actin stress fibers was also seen when RGC5 cells were induced to express Q368X and P370L mutant myocilins (Fig. 6C). The actin loss phenotype was not related to the Dox treatment as such treatment did not trigger any changes in the actin structure in parental RGC5 cells (Fig. 6D).

**Figure 7:**
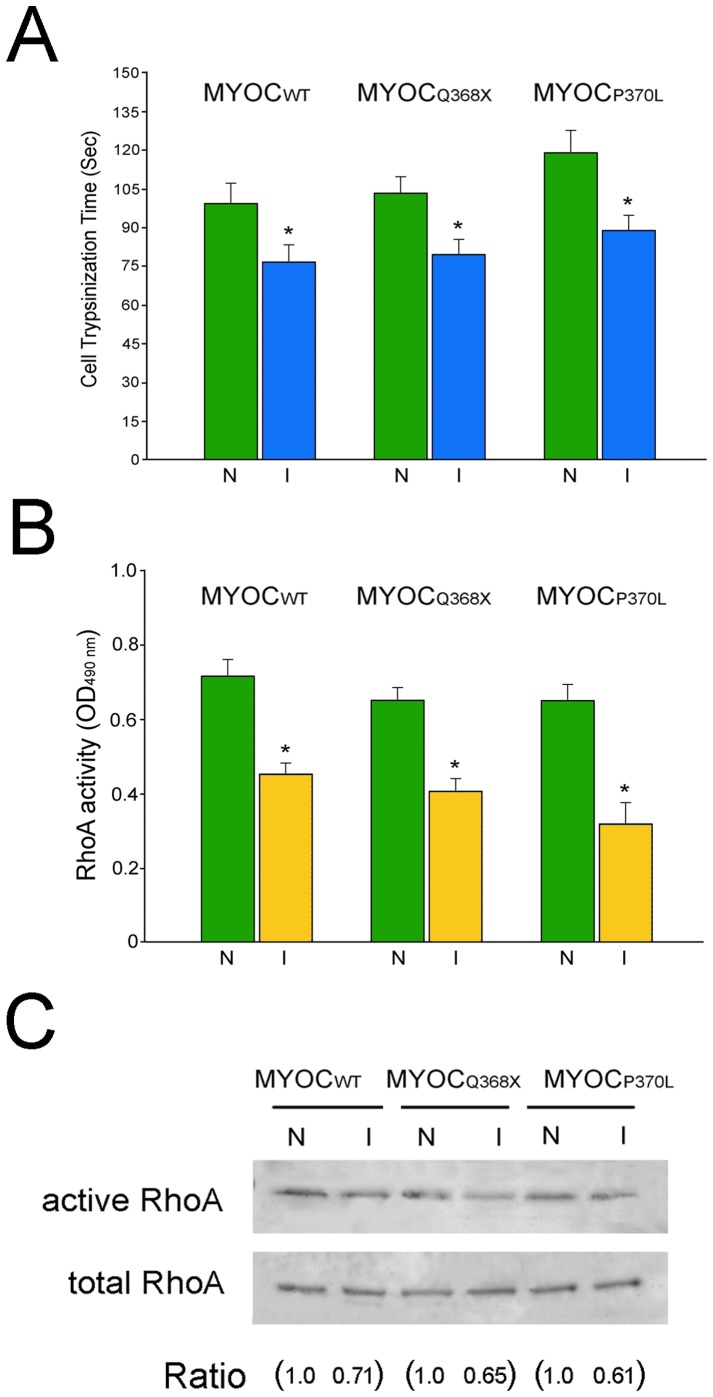
Trypsin sensitivity and active RhoA levels. **A.** Compared to non-induced (N) controls, the trypsinization time needed to round up the cells was reduced in induced (I) cells when moderate level of wild type (MYOC_WT_) or mutant (MYOC_Q368X_ and MYOC_P370L_) myocilin was expressed. **B**. Compared to non-induced (N) controls, active RhoA activity, measured by G-LISA RhoA assays, was lowered when wild type (MYOC_WT_) or mutant (MYOC_Q368X_ and MYOC_P370L_) myocilin was induced (I) to express in RGC5 cells. Results (mean ± SD) were analyzed by Student's t tests. *, P<0.001 compared to non-induced controls. **C**. Pull down assay showed a similar trend of active RhoA reduction as seen by G-LISA assay in **B**. Total RhoA was comparable between induced and non-induced samples. Ratios between active and total RhoA that were normalized to the respective non-induced controls are presented.

To further examine the cell-matrix adhesiveness, RGC5 inducible cells were subjected to trypsin sensitivity tests. The time needed for cells to round up was recorded using a confocal live cell imaging system and analyzed. It was found that RGC5 cells became more sensitive to trypsinization when induced (Fig. 7A). The trypsinization time for cells to round up was significantly (P*<*0.001) shorter for myocilin_WT_-, myocilin_Q368X_- and myocilin_P370L_-GFP-expressing cells than their respective non-induced counterparts (for wild type myocilin, non-induced [N]: 97±17 s; induced [I]: 78±15 s; for Q368X myocilin, N: 104±19 s, I: 81±18 s; for P370L myocilin, N: 116±29 s, I: 88±21 s, n = 20), indicating increased trypsin sensitivity or reduced cell-matrix adhesiveness.

G-LISA RhoA activation assays were carried out to measure the amounts of active RhoA in the cells. The level of GTP-bound or active RhoA in wild type or mutant myocilin-GFP-expressing RGC5 cells was approximately 35–50% lower (Fig. 7B, P<0.01) than that in non-induced controls (for wild type myocilin, non-induced [N]: 0.72±0.05, induced [I]: 0.46±0.03; for Q368X myocilin, N: 0.62±0.03, I: 0.41±0.03; for P370L myocilin, N: 0.64±0.05, I: 0.32±0.06, n = 2). Pull down assays for active and total RhoA were also performed (Fig. 7C). The active RhoA, relative to total RhoA level, was reduced by 30–40% in induced cells (Fig. 7C), corroborating the G-LISA results (Fig. 7B).

### Barrier function was reduced in wild type and mutant myocilin expressing cells

**Figure 8:**
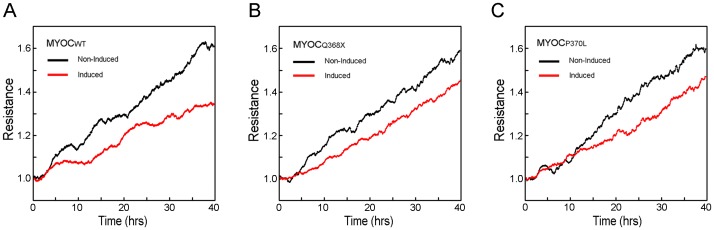
Barrier function in non-induced (black line) and induced (red line) RGC5 cells. The barrier function, evaluated by measuring the total electrical resistance (TER), was impaired in the moderate expressers. A representative TER experiment for each of the wild type (MYOC_WT_, **A**), Q368X (MYOC_Q368X_, **B**) and P370L (MYOC_P370L_, **C**) myocilin pairs is shown.

**Figure 9:**
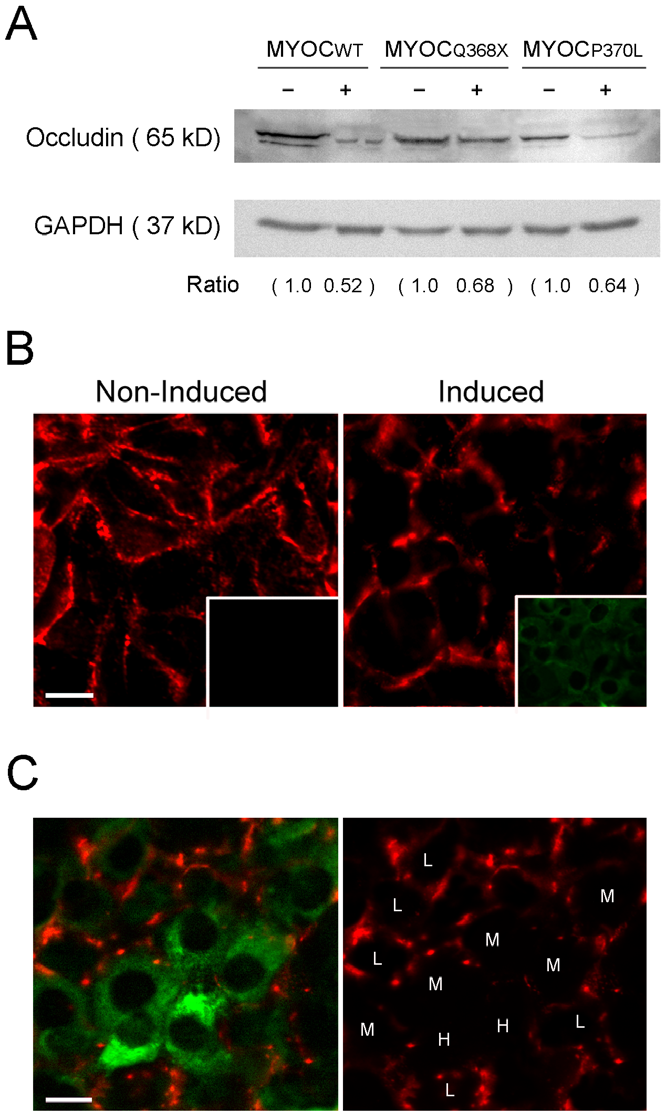
Western blotting and immunostaining for occluding levels. **A**. Lysates from non-induced (−) and induced (+) moderate expressers were examined by Western blotting. Ratios between occludin and GAPDH levels, relative to those in non-induced controls are presented for each of the myocilin wild type, Q368X, and P370L (MYOC_WT_, MYOC_Q368X_, and MYOC_P370L_) pairs. **B**. Non-induced RGC5 cells (left panel) and induced low expressers of wild type myocilin (right panel) were immunostained for occludin. Positive occludin staining in red was seen between cells around the border. The staining in induced cells was reduced compared to non-induced controls. Inset in the left panel shows negative control in which the primary antibody was replaced with normal rabbit IgG during the staining procedure. Inset in the right panel shows the same view to indicate the induced cells in green. C. Induced cells in a mixed culture of low (L), moderate (M), and high (H) expressers of wild type myocilin were stained for occludin. Compared to low expressers, moderate and high expressers with stronger green fluorescence had lower red fluorescence intensity for occludin. Scale bar, 10 µm.

The barrier function was evaluated by measuring the total electrical resistance (TER, also called transendothelial or transepithelial electrical resistance) using an electrical cell-substrate impedance sensing (ECIS) system. Dox-treated RGC5 cells that expressed myocilin_WT_-, myocilin_Q368X_- or myocilin_P370L_–GFP showed lower TER values than non-induced controls (Fig. 8), signifying decreased barrier function or increased permeability. The level of tight junction protein occludin was evaluated by Western blotting. Consistent with the reduced barrier function, the occludin level was approximately 30 to 50% lower in cells induced to overexpress myocilin_WT_-, myocilin_Q368X_- and myocilin_P370L_-GFP (Fig. 9A, the values relative to respective non-induced controls were 0.43±0.16 for wild type myocilin, P = 0.003; 0.68±0.02 for Q368X myocilin, P<0.001; and 0.60±0.20 for P370L myocilin, P = 0.036; n = 3). The occludin immunostaining in low expressers of wild type myocilin was also weaker than that in non-induced controls (Fig. 9B). The staining intensity was further diminished in moderate and high expressers in a low, moderate, and high expresser mixed culture (Fig. 9C).

## Discussion

Glaucoma is a group of chronic, degenerative optic neuropathies. Myocilin is one of the candidate genes linked to POAG, the most common form of glaucoma [29]. One of the risk factors for POAG is elevated intraocular pressure (IOP). The TM, a specialized tissue located in the chamber angle of the eye near the cornea, is responsible for regulation of the aqueous humor outflow and control of the IOP [30]. The TM tissue is composed of layers of trabecular beams that are made up of extracellular matrix elements [31]. TM cells that line the trabecular beams are believed to be important not only in the maintenance of normal TM function and homeostasis but also in the development of glaucomatous conditions.

Myocilin mutations are typically associated with high IOP cases [32]. This suggests that myocilin may have more of an impact on the cells in the aqueous outflow pathway such as the TM than those in the optic nerve head and the retina such as RGCs. Myocilin is found expressed in adult eyes in the RGCs, optic nerve axons and photoreceptors [16], [17], [33], [34], [35]. Its expression has been noted to increase in reactive astrocytes in the glial scar [35]. Wild type and mutant myocilins have also been shown to inhibit neurite outgrowth in RGC5 and PC12 cells [36]. Despite these observations however, it is still not clear whether myocilin has a functional role in RGCs. Neither the absence of myocilin in knockout mice [37] nor elevated levels of wild type mouse myocilin in the eye drainage structures of transgenic mice [38] led to IOP elevation or morphological changes in the retina. In transgenic mice expressing the Tyr437His mutant of human myocilin protein, the IOP was increased [39], [40]. RGC loss and axonal degeneration [39], [40] were also observed. The retinal changes on the other hand may not be related directly to the mutant myocilin transgene.

Our laboratory has performed studies on myocilin using cultured cells derived from normal human TM tissues [27], [41], [42], [43]. Several technical issues were encountered. First of all, fresh eyes from human donors are of limited availability. The TM tissue dissected from donor eyes is minute, containing limited number of cells and often resulting in poor yield of primary cultures. The proliferation potential of TM cells also diminishes generally with increasing post mortem time and age of the donors. Furthermore, the transfection efficiency of primary TM cells is very low (<10–15%), making it exceedingly challenging to conduct cellular and molecular biology investigations.

Progressive loss of RGCs and their exons is one of the hallmarks in POAG. In light of the potential impact of myocilin mutations on neuronal cells, and also due to the difficulties associated with cultured human TM cells, we undertook the current study, establishing inducible cell lines and extending our efforts to examine the consequences of myocilin and Q368X and P370L mutants on neuronal cells. RGC5, an immortalized RGC cell line used widely for various biological investigations [44], [45], was established by transforming postnatal day 1 rat retinal cells with E1A adenovirus [46]. It was however recently re-characterized as mouse, not rat origin by both mitochondrial and nuclear DNA analysis [47]. The cells were not positive for known markers of ganglion cells such as neurofilaments or Thy1.2. Nevertheless, they did stain positively for neuronal markers β-tubulin and PGP9.5, as well as for the microtubule-associated protein tau, and were able to differentiate with neurite extensions after treatment of staurosporine [44], [48] or trichostatin A [49], suggesting that these cells, while no longer a good model of RGCs, still represent neuronal precursor cells. They may still be suitable for molecular and cellular mechanistic studies of myocilin and be useful for future neuroprotection type of investigations.

Tet-on stable RGC5 cell lines were created to express wild type and mutant myocilin-GFP upon Dox induction. Three clones each with low, moderate, and high expression levels were obtained. Microscopic observation of low expresser of wild type myocilin-GFP revealed that it mimics the normal scenario with myocilin expression at the endogenous level while the high expressers represent overexpression situations displaying myocilin granules/aggregates in the cytosol of the cells (Fig. 2). The moderate and high expressers of myocilin_Q368X_- and myocilin_P370L_-GFP also showed prominent cytoplasmic aggregates (Figs. 3 and 4).

The endogenous, wild type myocilin in human TM cells is a secreted protein [7], [8], [32]. It undergoes an intracellular endoproteolytic processing in the central linker region by calpain II, producing two stable protein fragments, 35 and 20 kDa [50], [51]. This cleavage is speculated to regulate extracellular interactions of myocilin with proteins such as structural protein of lipid rafts, flotillin-1 [52], fibronectin [53], [54], laminin [19], [54], collagens [54], fibrillin-1 [54], optimedin [55], hevin [56] and SPARC [57], contributing thereby to the IOP control. Most of the myocilin mutations are located in the C-terminal olfactomedin-like domain (326–501 aa). These mutants, unlike the wild type protein, are not secreted and tend to aggregate inside the cells [23], [25], [32], [58]. Such secretion patterns were verified in our Tet-on inducible RGC5 cell lines. Upon Dox induction, myocilin_WT_-GFP was detected in both the culture medium (Fig. 2F) and the cell lysate while myocilin_P370L_- and myocilin_Q368X_ –GFP were only detected in the cell lysates, not in the culture media (Figs. 3F and 4F).

The function of wild type myocilin is still unclear. Data in the literature suggested that myocilin may play a role in cell-matrix interactions [20], [53], [59] and may inhibit neurite outgrowth [35], [36]. Using conditional medium containing myocilin, its proteolytic fragments or purified myocilin, Kwon et al. [60] noted that extracellular myocilin modulates Wnt signaling, affecting actin cytoskeleton organization that is essential for TM contractility and regulation of the IOP. When myocilin construct was transfected into human TM cells, however, the forced expressed protein within the cells provoked phenotypes that include elevation of the cAMP level, protein kinase A (PKA) activation, RhoA inactivation, and the ensuing loss of actin stress fibers, focal adhesions, matrix deposition, and cell-matrix adhesiveness [41].

With inducible RGC5 cell lines, it became feasible to determine whether myocilin phenotypes observed in TM cells were also replicated in neuronal cells. Contrasting the transfection situation, nearly 100% of the inducible cells are expressing the transgene upon induction, affording investigations of barrier functions and cell migration by scratch assays. Another advantage is the avoidance of myocilin overexpression by transfection using strong promoters that often lead to high amounts of forced expressed protein. Our Tet-on RGC5 inducible cells in addition offer useful model systems, since low, moderate as well as high expressers for both wild type and mutant myocilins are available. These clones would allow comparisons of pathologic phenotypes caused by transgene expression ranging from the endogenous to transfection/overexpression levels.

Similar to that found in TM cells in transfection studies [41], myocilin_WT_-GFP expression led to a loss of actin stress fibers (Fig. 6), a reduction of the cell-matrix adhesiveness as evidenced by trypsin sensitivity assay (Fig. 7A), and a decrease in the RhoA activity (Figs. 7B and 7C). Such alterations are similarly observed with myocilin_P370L_- and myocilin_Q368X_ -GFP mutants. The migratory activity was found to be impaired (Fig. 5), consistent with the demonstrated cytoskeletal alterations in both wild type and mutant myocilin-GFP-expressing RGC5 cells (Fig. 6).

With mutations in the olfactomedin-like domain of the myocilin sequence, Q368X and P370L mutants have been shown previously not to be secreted and exhibit gain-of-function effects [24], [25]. In the current study, these mutants were discovered to also produce nearly identical actin/cell adhesion/migration phenotypes as the wild type myocilin. Such findings suggest that the N-terminal sequence, rather than the C-terminal olfactomedin-like domain, is the key contributor to the myocilin phenotypes. Future sequence deletion experiments may help pinpoint the exact site(s) or motif(s) required.

Intracellular tight junctions provide structural integrity to epithelial and endothelial tissues. They regulate the passage of ions, water and molecules through the paracellular pathway, creating highly polarized barriers essential for homeostatic maintenance of the vertebrate physiological systems [61]. The tight junction barrier is composed of transmembrane proteins. Occludin, a 65-kDa membrane protein, is one of the crucial components that contribute to the tight junction stabilization and optimal barrier function [61]. Dysfunction of tight junction leads to increased paracellular permeability [62], [63].

In cell cultures, the tight junction integrity is typically measured using TER which represents the resistance of paracellular pathway rather than transcellular permeability. Therefore, the higher the TER, the lower is the permeability. As a positive control, we measured TERs in primary human TM cells without or with treatment of dexamethasone for 6 days. Results showed an increase in the TERs (data not shown), in agreement with previous reports that dexamethasone treatment enhanced the tight junction protein ZO-1 expression level, reduced the permeability and increased fluid flow resistance in human TM cells [64], [65]. In parallel experiments, induced expression of wild type and mutant myocilin in RGC5 cell lines resulted in reductions in the TERs (Fig. 8). The occludin level was concomitantly reduced (Fig. 9). These data suggest that the myocilin mediated permeability changes might be related to modifications in tight junctions. We have attempted to replicate these experiments using transfected primary human TM cells. However, due to the low transfection efficiency, the results were inconclusive.

In the retina, the retinal pigment epithelium (RPE) forms the outer blood retinal barrier and the endothelium of the retinal vessels constitutes the inner blood retinal barrier [66], [67]. The crossing of fluid and solutes through the blood retinal barrier is strictly controlled by tight junctions of the “non-leaky” type [67]. No other barriers have been described to date formed by either RGC themselves or RGCs with their surrounding cells. We stained normal rat retinal sections with an antibody against occludin. A strong staining was observed in the RPE layer as expected. In addition, the ganglion cell layer also positively stained (Fig. S1). The immunoreactivity was seen along the circumference of RGCs and/or cell-cell borders in the retinal ganglion layer, suggesting the presence of occludin in cell junctions. We propose that the retinal ganglion layer may form a “leaky” type of barrier. It will be of interest to investigate the function of this barrier and the change of barrier function during development or in response to pathological and pharmacological insults.

Signal molecules such as small guanosine triphosphatases (GTPases) of the Rho family are known to regulate cell adhesion, and cell-cell junctional assembly and disassembly [68]. The effect of RhoA activity on the barrier function is context and cell type dependent. Both negative and positive impacts have been reported. For instance, RhoA activation has been shown to lead to macrovascular endothelial barrier breakdown [68], [69] as well as cell-cell junction disruption, barrier destabilization and permeability increase in corneal endothelial cells [70], [71]. On the other hand, RhoA activation by cytotoxic necrotizing factor γ did not induce barrier breakdown in myocardial endothelial cells [72]. Very recently, inhibition of RhoA/ROCK pathway by ROCK inhibitor Y-27632 has been reported to decrease TER and increase permeability of Schlemm's canal cells [73]. Our data are consistent with the last finding that the myocilin phenotypes include both RhoA inactivation and barrier function reduction in RGC5 cells.

In summary, the Tet-on inducible RGC5 cells provide a new tool for exploring the effects of myocilin upregulation and mutations at cellular and molecular levels. Since the percentage of cells on the plate that would express the transgene upon induction approaches 100, studies including genomics, proteomics, and signal transduction are made possible. The consequences of a combination of myocilin mutation and stress such as hypoxia can also be examined utilizing the inducible cells.

## Materials and Methods

### Plasmids

A plasmid vector pTRE-MYOC-EGFP-INS-rtTA-IRES-hyg-pcDNA3.1z which contains both tetracycline regulatory and responsive components based on the Clontech's Tet-on advance system (Clontech, Mountain View, CA) was constructed (Fig. 1). The procedure of preparing construct pTRE-MYOC-EGFP-INS-rtTA-IRES-hyg-pcDNA3.1z was similar to that of pTRE-OPTN-EGFP-INS-rtTA-IRES-hyg-pcDNA3.1z described and reported earlier [74]. Briefly, to produce pTRE-MYOC-EGFP construct, MYOC-EGFP fusion gene was digested from pMYOC-EGFP and cloned into *EcoR I* and *BamH I* linearized pTRE-tight vector (Clontech). TRE-MYOC-EGFP was then shuttled to pBluescriptII-SK(+) vector and the insulator (INS) fragment was inserted to generate pBS-TRE-OPTN-EGFP-INS. Finally, rtTA-IRES-hygromycin fragment was digested from construct rtTA-IRES-hyg-pcDNA3.1z and ligated into pBS-TRE-OPTN-EGFP-INS [74]. Two other plasmid vectors containing mutation Q368X or P370L were similarly prepared.

### Antibodies

Rabbit anti-GFP and anti-RhoA antibodies were from Santa Cruz Biotechnology (Santa Cruz, CA). Horseradish peroxidase (HRP)- or Cy3-conjugated goat anti-rabbit and goat anti-mouse secondary antibodies were from Jackson ImmunoResearch (West Grove, PA). Anti-glyceraldehyde 3-phosphate dehydrogenase (GAPDH) antibody was obtained from Trevigen (Gaithersburg, MD). Rabbit polyclonal anti-occludin antibody was from Invitrogen (Grand Island, NY). Monoclonal anti-myocilin antibody [75] was kindly provided by Dr. Michael Fautsch at the Mayo Clinic, Rochester, MN.

### Cell cultures

Rat retinal ganglion RGC5 cells were obtained from the departmental core facility, deposited by Dr. Paul Knepper [76] generously provided and originally established [46] by Dr. Neeraj Agarwal (North Texas Health Science Center, Fort Worth, TX). RGC5 cells were grown in Dulbecco's modified Eagle's minimum essential medium (DMEM) supplemented with 10% fetal bovine serum and antibiotics. Normal human eyes from 44 and 49 years old donors were obtained from the Illinois Eye Bank (Chicago, IL). Human TM cells were cultured as previously described [27], [41], [43] on Falcon Primaria flasks in complete media containing DMEM, 10% fetal bovine serum, 5% calf serum and antibiotics. Dexamethasone (100 nM) treatment was carried out for 6 days as previously described [7].

### Establishment of Tet-on inducible myocilin-GFP-expressing RGC5 stable cell lines

RGC5 cells transfected with above-mentioned plasmid vectors were selected in hygromycin (100 µg/ml)-containing medium for approximately 2 weeks until colonies grew out. The cells were then trypsinized and induced with 1 µg/ml of Dox for 48 h. GFP positive cells that were sorted using DakoCytomation MoFlo into 96 well plates (1 cell/well) were incubated with maintenance medium (containing 50 µg/ml of hygromycin but without Dox) for another 2 weeks. Cells were screened for low, moderate, or high myocilin-GFP expressers after Dox induction by fluorescence microscopy. These expresser clones were allowed to multiply and were banked in liquid nitrogen.

### Western blotting

RGC5 inducible cells plated in T25 culture flasks were either induced for 48 h with Dox (1 µg/ml) to express myocilin_WT_-, myocilin_Q368X_-, or myocilin_P370L_-GFP, or non-induced as controls. Cells were harvested and lysed in the Cellytic buffer (Sigma, St. Louis, MO). Protein concentration was determined by Bradford assay (Pierce, Rockford, IL). Proteins in total cell lysates and culture media were resolved by sodium dodecyl sulfate-polyacrylamide gel electrophoresis (SDS-PAGE) and were subsequently transferred to nitrocellulose membranes for immunoblotting. Membranes were blocked with 5% non-fat milk in Tris-buffered saline with Tween 20 (TBST) for 1 h and incubated overnight with rabbit anti-GFP or mouse anti-myocilin to detect the expression level of myocilin-GFP fusion proteins, or with rabbit-occludin antibody to detect the level of occludin. Membranes were washed with TBST buffer 3 times and blotted with HRP-conjugated goat anti-rabbit or goat anti-mouse secondary antibody for 1 h. After further washing, the membranes were allowed to react with ECL (enhanced chemiluminescence substrate, Denville Scientific Inc. Metuchen, NJ) and the chemiluminescence signal was detected using the ImageQuant LAS4000 digital image system (GE Life Sciences, Piscataway, NJ).

### Actin staining and Immunofluorescence

RGC5 inducible cells were plated in 8-well chamber slides at 5000 cells/well. They were either induced for 48 h with Dox (1 µg/ml) or non-induced as controls. Actin filaments were stained with rhodamine-phalloidin (1∶50 in phosphate buffered saline or PBS, Invitrogen) per manufacturer's protocol. GFP fluorescence and actin staining in the cells were examined under a confocal laser scanning microscope (SP2 AOBS; Leica, Deerfield, IL). To further study the dose-dependence of the actin phenotype, low expresser was mix-cultured with moderate or high expressers of wild type myocilin-GFP, treated with Dox, and stained with rhodamine-phalloidin. Actin staining was also performed in parental (native) RGC5 cells following Dox treatment for 48 h. Untreated cells were used as controls. For occludin staining, non-induced cells, induced low expressers, and induced cells in a mixed culture of low, moderate, and high expressers were fixed in 4% paraformaldehyde and incubated with rabbit anti-occludin and Cy3-goat anti-rabbit IgG.

### *In vitro* scratch assay

RGC5 inducible cells from moderate clones were plated in 6 well plates and induced for 48 h. Cells were serum starved for 2 h and treated with 5 µg/ml of mitomycin C for 2 h [26]. Each well in confluence was scratched gently using a disposable 10 µl pipette tip to generate a confined scratch area [26], [27]. The cells were incubated in serum-free media in the presence of mitomycin C (to block cell proliferation). The ability of cells to migrate into the scratched areas was monitored 11 h later by differential interference contrast and fluorescence microscopy (Zeiss Axiovert100M inverted microscope, Thornwood, NY) and documented by photography. The total area of the scratch and the area covered by the cells within the scratch in each 10× field were measured with Image J software. A total of 8 fields were analyzed and the percentage of areas covered by migratory cells in each sample was calculated. Experiments were repeated 3 times and the statistical significance of the data was determined by Student's t tests.

### Trypsinization sensitivity assay

RGC5 inducible cells (moderate expressers) were plated in poly-D-lysine coated 35 mm glass bottom dishes (MatTek, Ashland, MA). After Dox induction, the cells were washed with PBS and Versene. Following addition of 0.125% trypsin-EDTA solution, morphologic changes were monitored and recorded every 10 s for a total of 5 min using a confocal spinning disc live cell imaging system (Carl Zeiss). The time needed for the cells to round up was analyzed [41] from the captured images. Experiments were repeated 3 times.

### Active RhoA activities

RGC5 inducible cells either induced with Dox (1 µg/ml) for 48 h or non-induced were serum-starved for 18 h and lysed. G-LISA® RhoA activation assay Biochem Kit™ (absorbance based) (Cytoskeleton Inc., Denver, CO) was used to determine the active, GTP-bound RhoA per manufacturer's instruction. The absorbance at 490 nm from wild type or mutant myocilin-GFP-expressing cells (mean ± SEM) was compared with their respective non-induced controls. Experiments were repeated 2 times.

Active RhoA was also measured with pull down assay (Rho activation kit, Cytoskeleton) as previously described [41]. Non-induced and induced RGC5 cells serum starved for 18 h were lysed. The lysates were mixed at 4°C with GST-Rhotekin bound to Sepharose beads for 1 h. The proteins bound to the beads were resolved by 12% SDS-PAGE and immunoblotted with anti-RhoA. Cell lysates preincubated with GTPγ S and GDP served as positive and negative controls respectively. Prior to incubation with the beads, aliquots were removed from samples for total RhoA. Amounts of active GTP-RhoA bound to GST-Rhotekin were normalized against amounts of total RhoA in the cell lysates.

### Barrier function

The cell barrier properties were measured using an ECIS system (ECIS1600R; Applied Biophysics, Inc., Troy, NY) [77], [78]. Briefly, 20,000 RGC5 inducible cells were plated in each well of 8W10E+ array (8 polycarbonate wells each containing two sets of 20 circular 250 µm diameter active electrodes located on inter-digitated fingers to provide measurements of cells upon a total of 40 electrodes). Cells were either induced with Dox or non-induced as a control. The culture medium was used as the electrolyte. The TER was measured dynamically across the monolayer for another 40 h. As a control, human TM cells (passage 3) cultured as previously described [27], [41] were plated in the 8W10E+ array and treated with dexamethasone (100 nM, Sigma) for 6 days. The TER was measured for another 20 h.

## Supporting Information

Figure S1 Immunostaining of rat retinal section for occludin. The section was stained with polyclonal rabbit anti-occludin antibody (left panel, in red). Hematoxylin and eosin staining (right panel) was performed on a serial section to demonstrate retinal layers. Retinal pigment epithelial (RPE) and retinal ganglion cell (RGC) layers showed strong staining of occludin. Scale bar, 100 µm.
(TIF)
