# Supplementary figures and images for: Establishment of Inducible Wild Type and Mutant Myocilin-GFP-Expressing RGC5 Cell Lines
Source: PLoS One. 2012 Oct 17;7(10):e47307. doi: 10.1371/journal.pone.0047307 (PMC3474840; doi:10.1371/journal.pone.0047307)

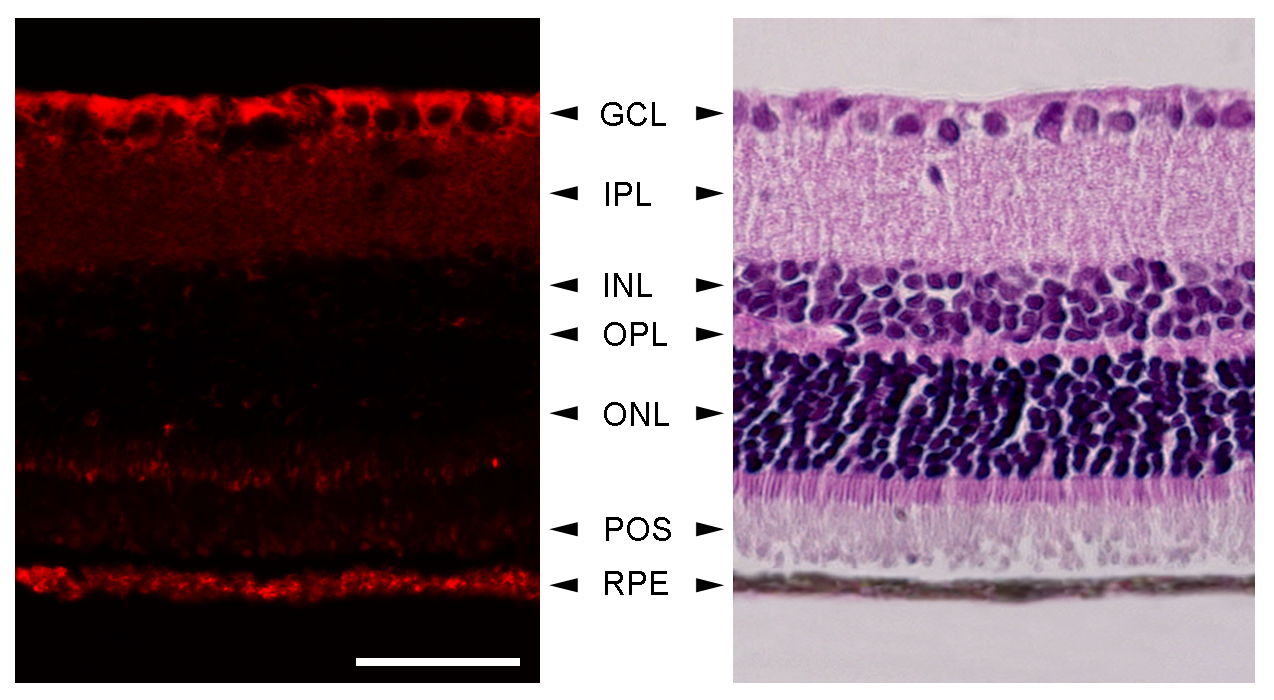

Supplement: Figure S1 — Immunostaining of rat retinal section for occludin. The section was stained with polyclonal rabbit anti-occludin antibody (left panel, in red). Hematoxylin and eosin staining (right panel) was performed on a serial section to demonstrate retinal layers. Retinal pigment epithelial (RPE) and retinal ganglion cell (RGC) layers showed strong staining of occludin. Scale bar, 100 µm. (TIF) [file pone.0047307.s001.tif]
